# The influence of sequential drilling and machining parameters on uncut fiber formation and delamination damage in fiber-reinforced composites

**DOI:** 10.1038/s41598-026-40786-y

**Published:** 2026-02-21

**Authors:** Sayed Mohammad Hossein Izadi, Ali Mozaffari

**Affiliations:** 1 Aerospace Department, Shahid Sattari Aeronautical University of Science & Technology, Tehran, Iran; 2 Department of Aerospace Engineering, K. N. Toosi University of Technology, Tehran, Iran

**Keywords:** Sequential drilling, Machining parameters (feed & spindle speed), Delamination, Uncut fibers, Composite materials, Engineering, Materials science

## Abstract

Drilling is crucial in composite manufacturing, but often induces delamination and uncut fibers, degrading structural integrity. This study examines the combined influence of sequential drilling (using drills of progressively increasing diameter) and machining parameters (feed rate and cutting speed) on damage in fiberglass composites. Sequential drilling was performed at a feed rate of 1000 mm min⁻¹ and spindle speed of 500 rpm, while the effects of machining parameters were investigated over feed rates ranging from 50 to 2000 mm min⁻¹ and spindle speeds from 500 to 3000 rpm. Results show that sequential drilling effectively reduced delamination at both the entrance and exit of drilled holes, while simultaneously decreasing exit-side uncut fibers and increasing entrance-side uncut fibers. Furthermore, increasing cutting speed significantly mitigated damage, while higher feed rates worsened it. Three-point bending tests revealed that modified drilling conditions increased the fracture load of drilled specimens by up to approximately 18%, with fracture loads rising from about 956 N to 1130 N depending on feed rate and spindle speed. Unlike previous studies that primarily examine drilling strategies or machining parameters in isolation, this work provides a unified experimental framework that links sequential drilling methodology, quantitative damage characterization (via image processing), and mechanical performance (via three-point bending tests). Quantitative damage assessment reveals the complex interplay between drilling method and parameters, offering valuable insights for optimizing composite drilling processes.

**Supplementary Information:**

The online version contains supplementary material available at 10.1038/s41598-026-40786-y.

## Introduction

Drilling serves as a fundamental process in the manufacturing of composite structures, playing a crucial role in industries such as aerospace and automotive, where precision and integrity are paramount. As the demand for lightweight and high-strength materials increases, fiber-reinforced composites have emerged as a popular choice due to their superior mechanical properties and efficiency^1–4^. However, the unique characteristics of these materials pose significant challenges during machining operations.

One of the most critical issues encountered during the drilling of composites is damage, particularly delamination and uncut fibers. Delamination occurs when the layers of composite materials separate, leading to a reduction in structural integrity and performance^5^. Uncut fibers, on the other hand, can compromise the load-bearing capabilities of the material and affect the overall durability of the component^6,7^. As such, it is essential to explore effective drilling methods that minimize these detrimental effects.

Tsao et al.^8^ highlighted the challenges of characterizing delamination, due to irregular damage patterns. They proposed a novel equivalent delamination factor (${F_{ed}}$) using a core-saw drill, designed to reduce chip removal and delamination, and compared its effectiveness to existing methods (${F_a}$ and ${F_{da}}$). Their findings suggest ${F_{ed}}$ is a suitable metric for quantifying delamination in CFRP drilling.

Voß et al.^9^ discussed the evaluation of bore exit quality in carbon-fiber-reinforced plastics (CFRP) by examining various delamination factors, including the original delamination factor and its adjusted versions. They proposed a new quality function (${Q_d}$) based on five damage indicators to provide a more reliable assessment of bore quality, particularly for unidirectional carbon fiber-reinforced polymers (CFRPs). Their approach was supported by drilling tests that compared ${Q_d}$ with traditional measures of tool wear and thrust force increase.

Baraheni and Amini^10^ explored the machining challenges of CFRPs and evaluated the impact of nano-graphene incorporation and various machining parameters, including feed rate and tool type, in both conventional and ultrasonic-assisted drilling. Their findings indicate that a lower feed rate with an HSS-8% cobalt tool significantly improves hole quality, including delamination and roundness, especially when ultrasonic vibration is applied.

Xu et al.^11^ examined the drilling-induced defects in carbon fiber reinforced polymer composites, such as burrs, tearing, and delamination, and proposed adjusted damage criteria for quantifying these defects. Their study involved drilling tests on T800/X850 CFRP laminates with different drill types and utilized advanced imaging techniques to evaluate drill performance and the effectiveness of the proposed criteria in assessing hole quality. Jabbaripour et al.^12^ developed a polypropylene composite reinforced with carbon fibers and calcium carbonate nanoparticles, finding significant improvements in tensile, bending, and impact strengths. Their high-speed drilling tests revealed that optimal dimensional and geometrical tolerances, minimal uncut fibers, and reduced delamination defects occur at the highest spindle speeds and lowest feed rates, highlighting the importance of these parameters in achieving superior hole quality and surface finish.

Barik et al.^13^ investigated drilling-induced defects in bi-directional woven carbon fiber–reinforced plastics using a full factorial design and response surface methodology. They found that high feed rate and large drill point angle increased delamination, while higher spindle speed with low feed reduced surface roughness and circularity errors, correlating these damages with thrust force and torque variations.

In another study, Barik and Pal^14^ compared the drilling performance of TiAlN-, TiN-coated, and uncoated drills in CFRP laminates, analyzing thrust and torque signals through wavelet methods. They found that feed rate strongly influenced delamination, while coated drills produced more uniform torque and better hole quality, with torque wavelets serving as reliable indicators of surface integrity. Also, in another study^15^, the authors examined tool wear evolution during drilling of woven CFRP using uncoated, TiAlN-, and TiN-coated carbide drills under constant conditions. They observed that tool wear increased with hole number, leading to higher thrust, delamination, and roughness, while TiN-coated drills offered superior durability, and low-frequency force and torque wavelets effectively indicated wear and hole quality.

Pervaiz et al.^16^ investigated the effects of hole inclination, lubrication methods, tool coating, and drill geometry on the drilling quality of CFRP laminates. They found that thrust forces increased significantly with inclined holes in dry cutting, while using compressed air lubrication minimized this increase to the lowest level. The study highlighted the importance of maintaining hole integrity for the overall service life.

Tian et al.^17^ proposed a simple and efficient method to determine an n-segment constitutive law for simulating R-curve behavior in delamination growth of composite laminates, driven by fiber bridging. Their approach, implemented in finite element software through a user-defined subroutine, demonstrated robust agreements between experimental and predicted load–displacement responses across varied material systems, requiring only load and displacement data without the need for parameter adjustments.

Izadi et al.^18^ developed a new mixed mode fracture criterion that accounts for damage created in functionally graded materials (FGMs), including voids and holes formed in the fracture process zone due to loading that exceeds the allowable limits. The criterion was then compared to experimental results and demonstrated good agreement. In another study^19,20^, the authors identified several fiber bridging mechanisms through microscopy, showing that fiber volume fraction and interfacial friction strongly influence crack resistance and energy dissipation. Their findings highlighted that the fracture behavior of FGMs must be evaluated by considering the interactive effects of all layers rather than analyzing each layer separately.

Gao et al.^21^ studied machining damage in fiber-reinforced composites, focusing on milling and grinding processes. They analyzed delamination, burr, and tear formation mechanisms in milling and explored material removal, thermal effects, and surface integrity in grinding. The authors also studied damage suppression strategies, including ultrasonic vibration, cryogenic cooling, and minimum quantity lubrication (MQL), highlighting their effectiveness in reducing machining forces and temperatures.

Wang et al.^22^ investigated the impact of horizontal ultrasonic vibration in rotary ultrasonic machining (RUM) of CFRP composites, focusing on its effect on cutting force. They developed a novel mechanistic model based on brittle fracture, successfully predicting cutting force trends consistent with experimental data. This model provides a foundation for predicting other RUM process parameters in CFRP grinding.

Qiu et al.^23^ investigated the influence of stepped drill geometry and machining parameters on CFRP drilling, focusing on cutting force and hole wall damage. Their experiments comparing twist drills and stepped drills revealed that a specific stepped drill geometry minimized thrust force, reducing delamination risk. The study identified drill geometry, feed rate, and spindle speed as key factors affecting hole wall damage, introducing a novel cutting force metric to quantify this relationship.

Despite the extensive body of research on composite drilling, several limitations remain in the existing literature. Most prior studies focus on the influence of machining parameters such as feed rate, spindle speed, tool geometry, or coating independently, without systematically considering their interaction with alternative drilling strategies such as sequential drilling. In addition, while delamination has been widely investigated as a primary damage mode, the formation of uncut fibers, particularly its asymmetric behavior at the hole entrance and exit, has received comparatively limited attention. Moreover, many studies rely solely on geometrical or force-based damage metrics, with limited efforts to directly correlate drilling-induced damage with post-machining mechanical performance. As a result, a comprehensive understanding that simultaneously links drilling methodology, machining parameters, multiple damage mechanisms, and structural performance remains lacking, especially for fiberglass composite systems.

Building upon these gaps, this study focuses on the combined effects of sequential drilling methodologies and vital machining parameters, specifically feed rate and spindle speed, on the damage formation in fiberglass composites. This investigation evaluates sequential drilling with progressively increasing drill diameters and analyzes the impact of machining parameters on the incurred damage. These analyses provide valuable insights into modifying drilling techniques for composite materials. To quantify this damage, image processing techniques will be used to analyze the hole geometry, specifically focusing on the presence of uncut fibers and delamination at the hole entrance and exit. Furthermore, three-point bending tests (3 PB) will be employed to determine the fracture load of the drilled specimens, directly correlating the observed damage (uncut fibers and delamination) with the resulting mechanical performance. This work is necessary because while previous studies have examined the impact of machining parameters individually, a comprehensive understanding of their combined effects of sequential drilling and machining parameters on damage in fiberglass composites remains lacking. This study addresses this gap by providing a holistic investigation linking drilling methodology, machining parameters, damage quantification (via image processing), and mechanical performance (via three-point bending).

## Experimental procedure

**Fig. 1:**
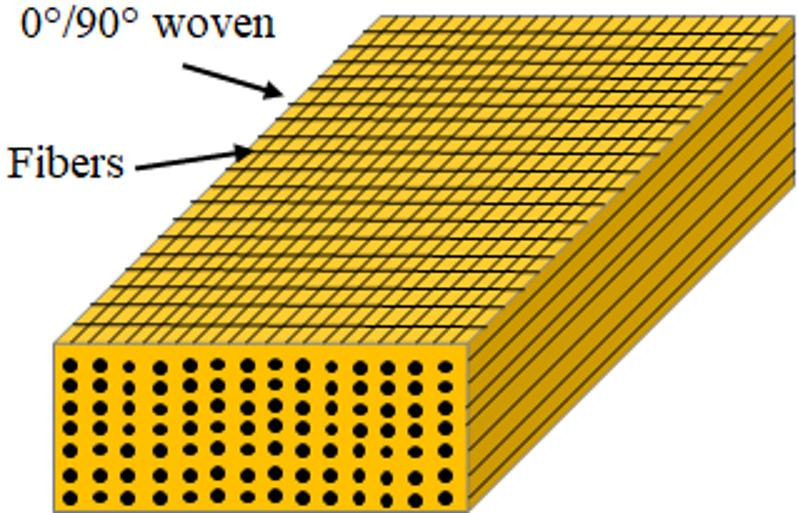
Schematic illustration of the stacking sequence and alternating fiber orientations (0°/90°) in the seven-layer woven fiberglass composite laminate.

**Fig. 2:**
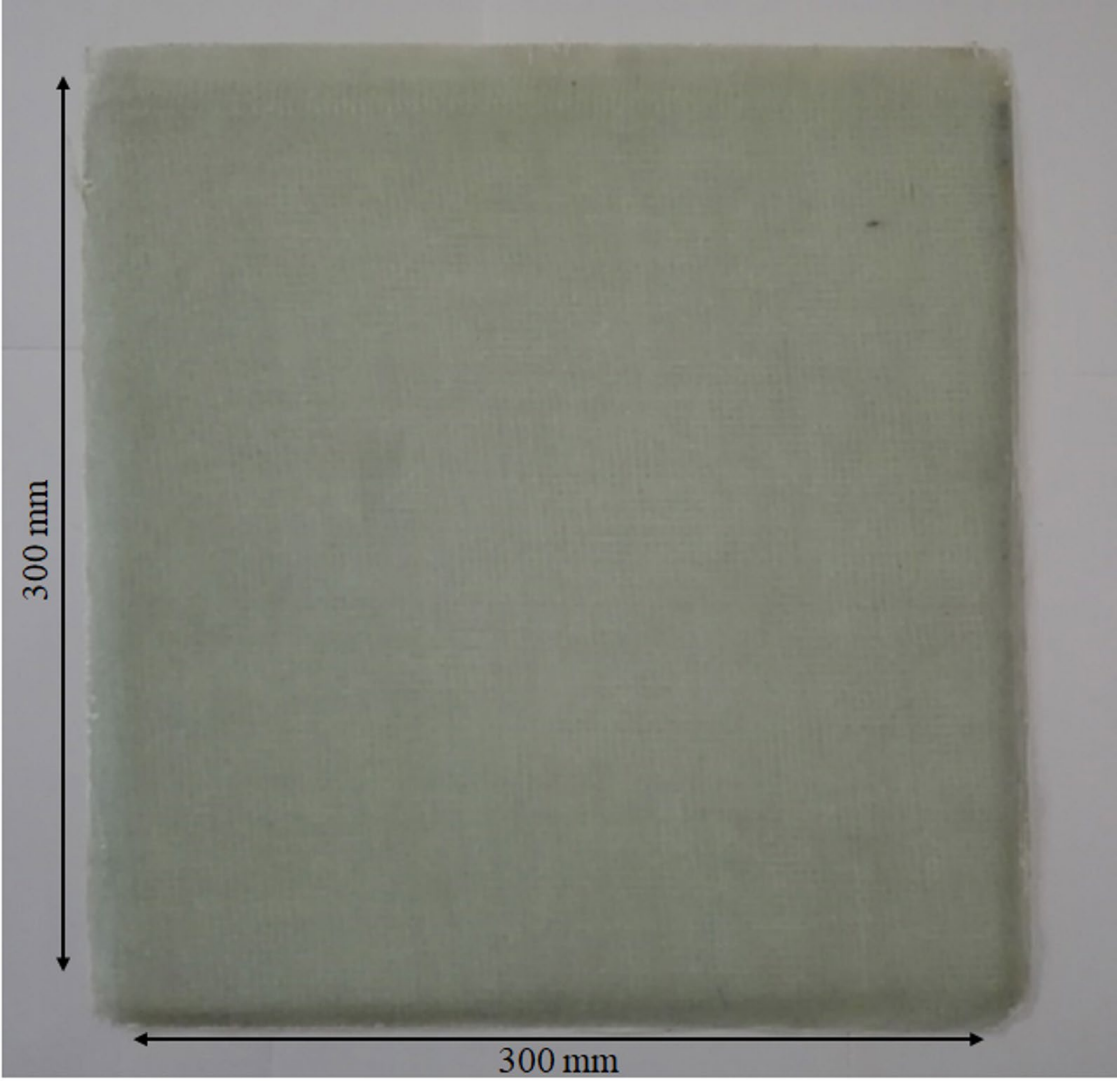
Fabricated composite laminate with hand lay-up method.

A composite laminate with dimensions 300 mm × 300 mm × 4.54 mm was fabricated using the vacuum-assisted hand lay-up method. The laminate consisted of seven layers of 300 g/m² glass woven fabric, arranged in a symmetric configuration with alternating 0° and 90° fiber orientations to provide balanced in-plane stiffness and improved dimensional stability during curing. The stacking sequence is illustrated in Fig. 1, which shows the alternating fiber orientations across the laminate thickness. Epoxy resin 828, combined with the appropriate hardener, was used as the matrix material to ensure strong adhesion and effective stress transfer between fibers. Each woven fabric layer was carefully aligned and impregnated with resin to achieve uniform wetting and minimize air entrapment. Subsequently, the laminate was subjected to vacuum pressure during the curing process to extract entrapped air and excess resin, thereby reducing void content, improving fiber–matrix consolidation, and ensuring consistent laminate thickness. The laminate was cured at room temperature for 48 h under vacuum-assisted pressure to achieve good consolidation and surface uniformity. The final fabricated composite laminate is shown in Fig. 2, demonstrating the smooth surface quality and uniform thickness achieved after curing.

In order to prepare the required specimens for drilling, several specimens with the dimensions of 280 × 40 × 4.54 mm were cut from the fabricated composite laminate.

The experiments for this research were designed using a multi-factor experimental design method. To increase the accuracy of the tests, all experiments were repeated three times. To gain better control over the drilling process, the tests were conducted using a T.M.V-850All CNC milling machine, which has a maximum power of 7.5 to 9 $kW$.

**Fig. 3:**
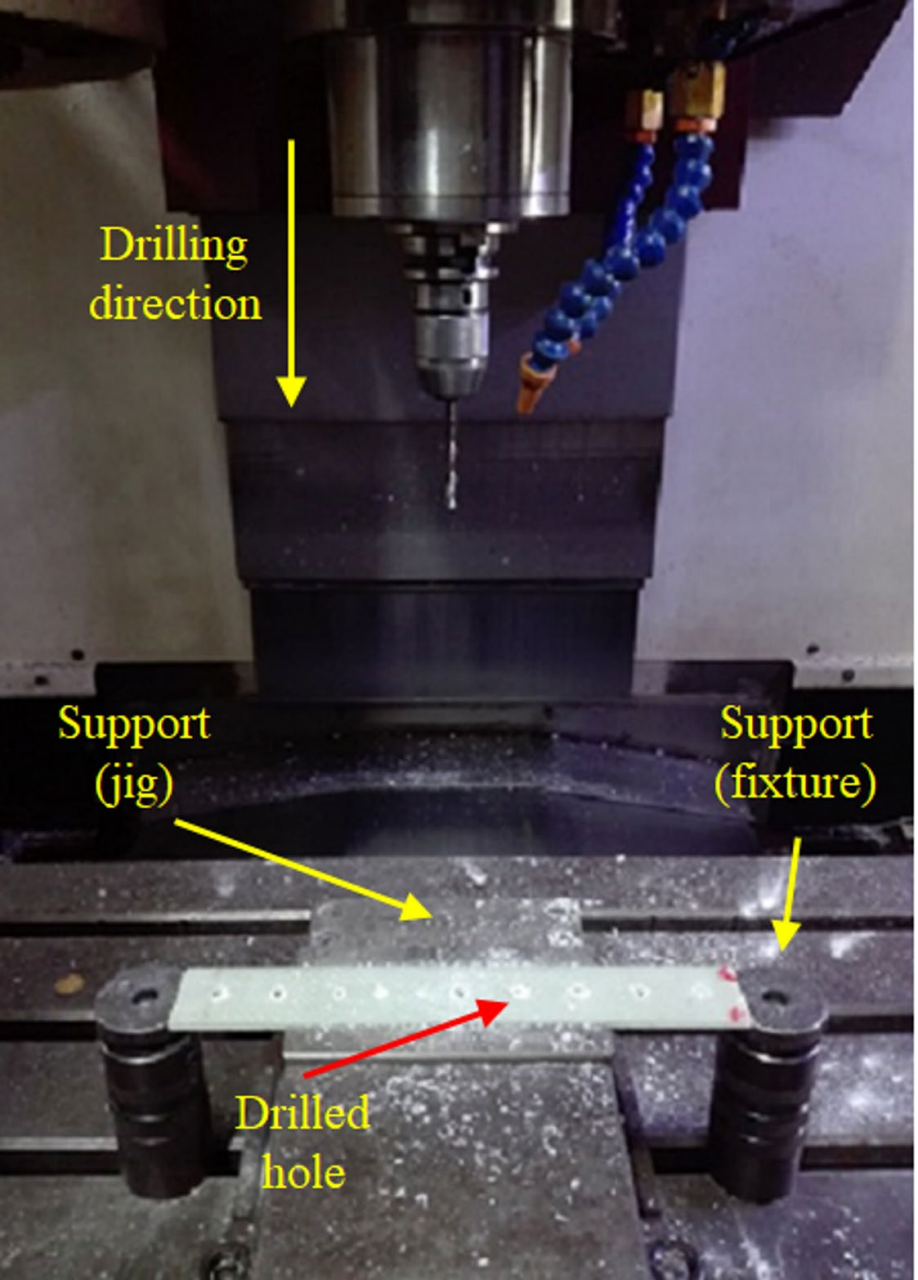
Drilling process of the specimens.

Various drill bits with diameters of 3, 5, 7, and 10.1 mm were used to conduct the sequential drilling process for different hole sizes. All twist drill bits were made of high-speed steel (HSS) according to the DIN 338 standard. The diameters of 7 mm and 10 mm were designated as the final and target diameters for the drilled holes. To investigate the effect of the number of drill bits on the damage caused to the composite parts, experiments were designed to commence drilling operations using drills with diameters approximating 30%, 50%, and 70% of the required hole diameter, followed by a drill with a diameter equal to the final size of the hole. The drilling process has been depicted in Fig. 3.

As mentioned in Table 1, holes with final diameters of 7 mm and 10.1 mm were created using three different drilling methods. Method 1 involved a single drilling operation to the final diameter. Method 2 used two drills of progressively increasing diameters to achieve the final diameter, and Method 3 employed three such drills. The feed rate and spindle speed (cutting speed) were set at 1000 mm min^− 1^ and 500 rpm, respectively. This selection was informed by the CNC machine’s specifications and established industrial practices for comparable applications.

Drill bits were replaced with new ones after every four drilling operations to mitigate the influence of tool wear on the results. The use of coolant was avoided to eliminate the possibility of unwanted chemical reactions between the coolant and the workpiece material.

**Table 1:** Designated parameters for sequential drilling tests.

Number of tests	Final hole diameter (mm)	Number of drills used	Drill bit diameter (mm)	Feed (mm min^− 1^)	spindle speed (rpm)
1	7	1	7	1000	500
2	7	2	3–7	1000	500
3	7	3	3–5-7	1000	500
4	10.1	1	10.1	1000	500
5	10.1	2	5–10.1.1	1000	500
6	10.1	3	3–7-10.1	1000	500

Feed rate refers to the speed at which the cutting tool advances into the workpiece, typically measured in mm min^− 1^. Spindle speed (cutting speed) represents the rotational speed of the drill bit, measured in revolutions per minute (rpm).

To determine the impact of varying parameters on the drilling process, tests were conducted using two drill bits with diameters of 7 mm and 10.1 mm. For each test, the feed rate was set at three distinct levels: 50 mm min^− 1^, 1000 mm min^− 1^, and 2000 mm min^− 1^. Additionally, spindle speeds were varied among three settings: 500 rpm, 1500 rpm, and 3000 rpm. To comprehensively study the effects of feed and spindle speed during the drilling process, the experiments followed a systematic approach. For each drill bit diameter, the spindle speed was initially kept constant at 1500 rpm while the feed rate was varied to observe its effects on drilling performance. Subsequently, the feed rate was fixed at 1000 mm min^− 1^, allowing for an examination of how changes in spindle speed influenced the drilling process. All drilling procedures adhered strictly to the methods employed in the sequential drilling tests to ensure consistency and reliability of the data collected.

**Fig. 4:**
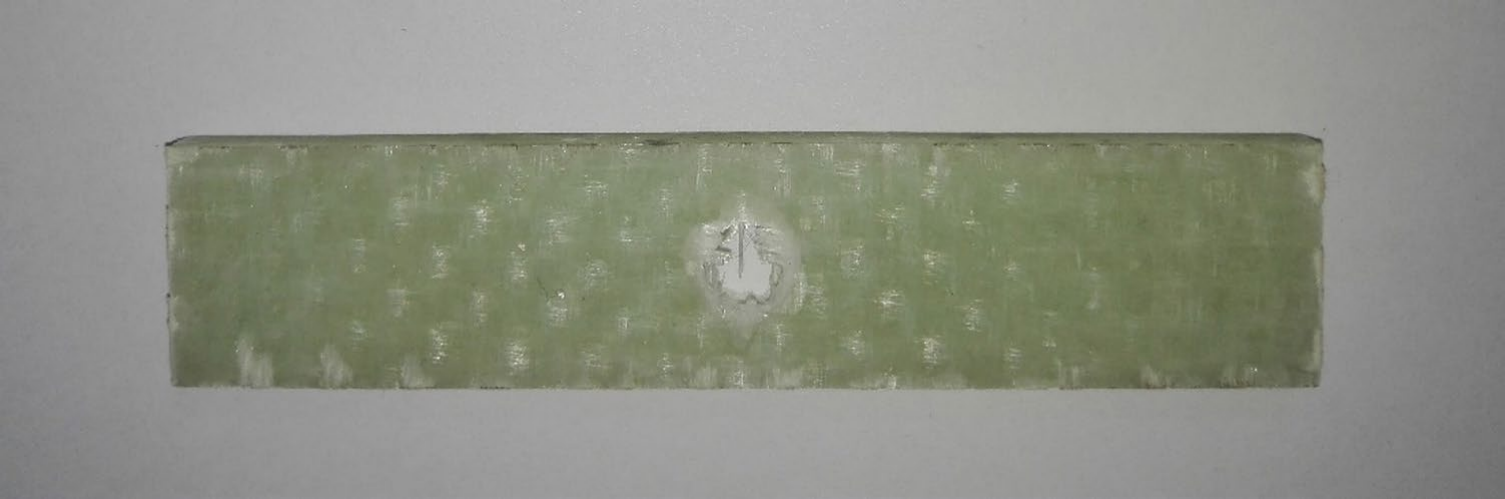
Prepared specimens with dimensions of 88 × 18.2 × 4.54 mm and an embedded 7 mm drilled hole.

To better study the effects of feed rate and spindle speed on the integrity and quality of the final product during the drilling process, a three-point bending test was utilized. This test allowed for an exploration of the impact of damage incurred during drilling on the stiffness of the specimens and the required thrust force to break them. Based on ASTM D790^24^, several specimens measuring 88 × 18.2 × 4.54 mm were cut from the fabricated composite laminate, and a hole was created in each specimen using a 7 mm drill bit. The drilling process followed the same protocol as previously described, where for three specimens, the feed rate was held constant while the spindle speed was varied; conversely, for the remaining three specimens, the spindle speed was fixed and the feed rate was changed. Figure 4 shows the hole created in one of the specimens prepared for the three-point bending test.

**Fig. 5:**
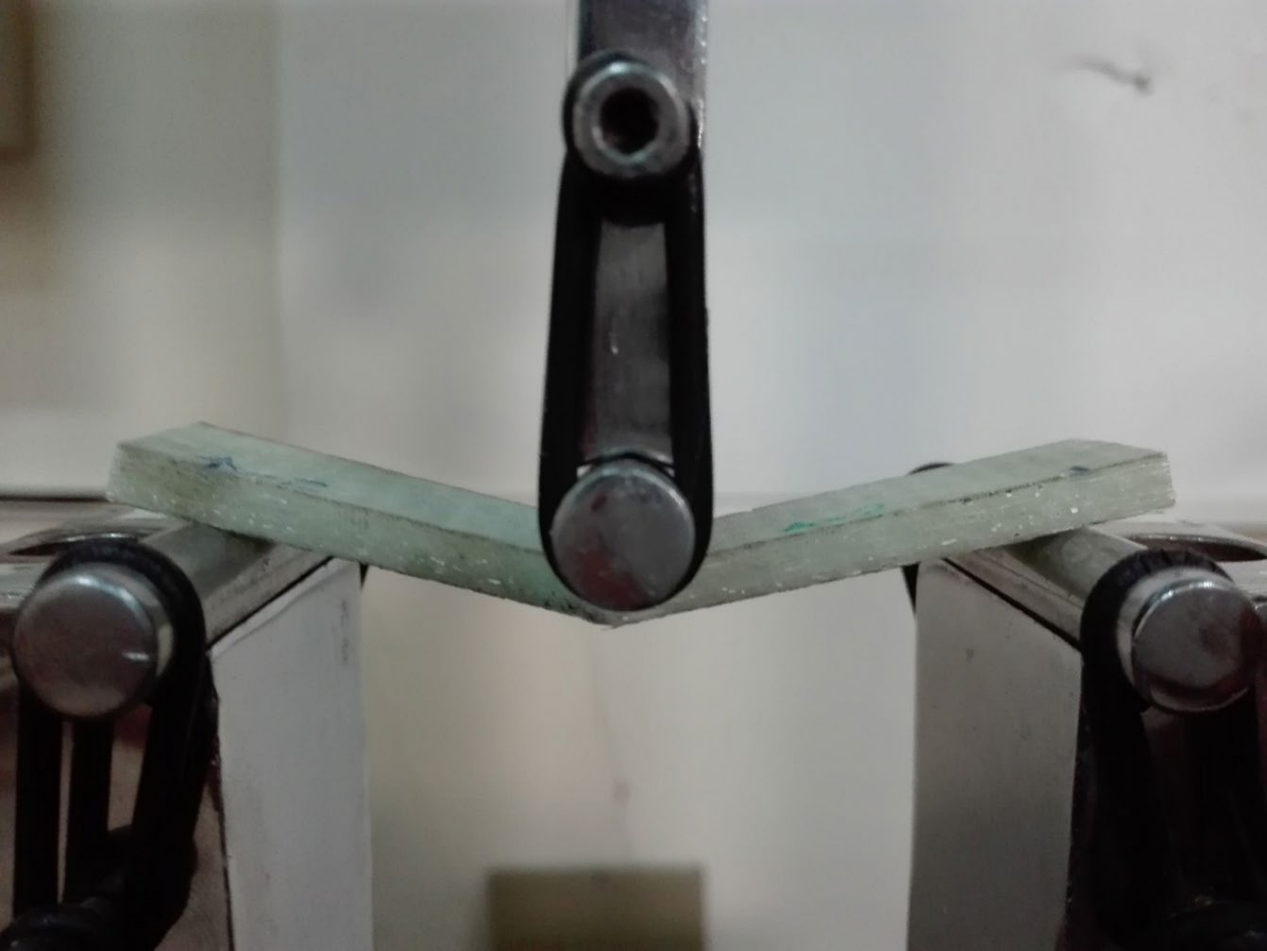
Applying three-point bending loading on the drilled specimens.

The SANTAM STM-150 universal testing machine was employed to apply bending loads on the prepared specimens, and all tests were repeated three times to enhance the accuracy of the results. The three-point bending tests were conducted at room temperature using a support span length of 58 mm, corresponding to a hangover length of 15 mm on each side of the specimen, and a constant crosshead displacement rate of 0.5 mm min⁻¹, in accordance with ASTM D790. Load was applied at the mid-span through a cylindrical loading nose, while the specimens were simply supported by two cylindrical rollers. Figure 5 illustrates the process of the three-point bending test on damaged specimens.

## Image processing

Following the completion of all experimental procedures, the drilled specimens were subjected to digital imaging and quantitative analysis using ImageJ software, a widely recognized platform for microstructural image processing. High-resolution images of each drilled hole were captured under uniform lighting conditions to ensure consistent image quality. Prior to analysis, the images were converted to grayscale, and contrast enhancement was applied to emphasize the boundary between the intact matrix and damaged regions.

The distinction between delaminated regions and uncut fiber areas was achieved through pixel-intensity thresholding and edge detection. Delaminated regions were identified as areas exhibiting separation or lifting of layers, visible as distinct tonal discontinuities around the hole edge, while uncut fiber regions appeared as continuous fiber bundles protruding beyond the intended hole boundary without layer separation. Threshold parameters were adjusted uniformly for all specimens.

**Fig. 6:**
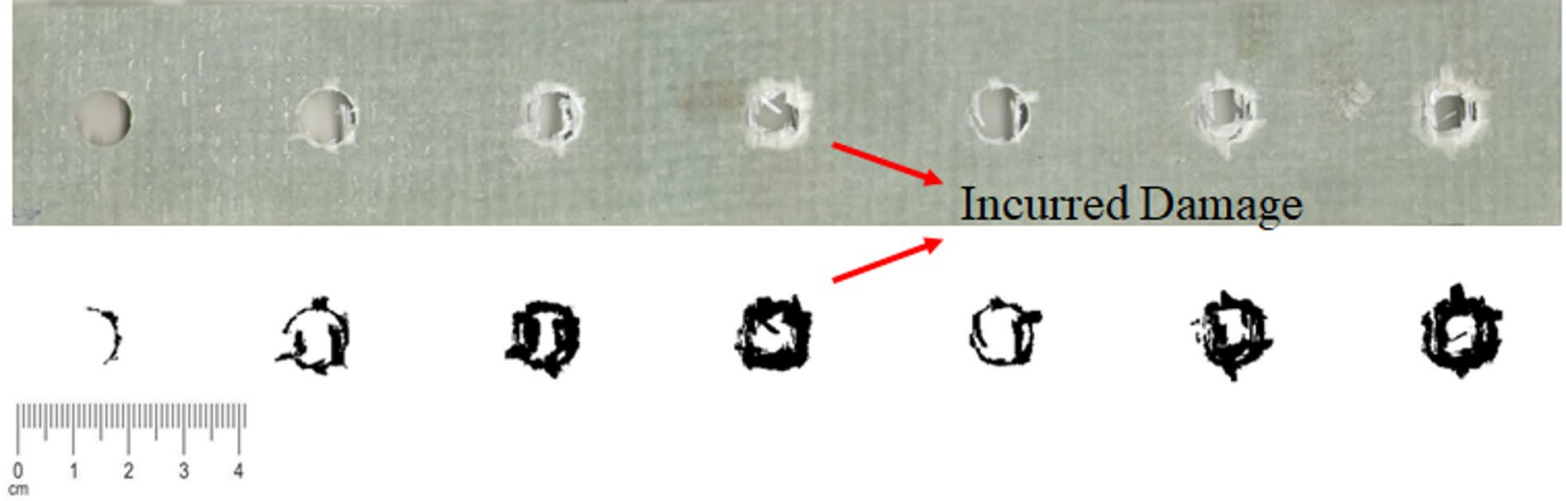
Image analysis process for the specimen with a final hole diameter of 10.1 mm.

To verify reproducibility, several randomly selected images were reprocessed under identical threshold settings, confirming that variations in lighting or contrast had minimal influence on the quantified damage indices. This consistent segmentation approach provided reliable comparisons of delamination and uncut fiber areas across different drilling conditions. Figure 6 illustrates the image analysis process for a specimen with a final hole diameter of 10.1 mm, showing the contrast enhancement, thresholding, and boundary delineation steps used to extract damage metrics.

As illustrated in Fig. 6, the areas of the material exhibiting delamination and uncut fibers are clearly distinguishable from the rest of the specimen. These damaged regions were highlighted by changing their color to black. This enabled accurate quantification of the damaged area using ImageJ’s measurement tools. Furthermore, since uncut fibers typically form within the hole area while delamination occurs around the hole, these two types of damage are separable and easily recognizable.

Special criteria are needed to determine the level of failure and damage that happened during the machining of composites; So far, several evaluation criteria have been introduced to evaluate the drilling quality of composites in terms of delamination damage; Among them, the equivalent delamination factor ${F_{ed}}$, developed by Tsao et al.^8^ has a higher accuracy due to the use of the exact area of the delaminated area around the hole (${A_d}$) in its formulation (Eq. (1)).

1 $${F_{ed}}=\frac{{{D_{_{e}}}}}{D},\,\,\,\,\,\,\,{F_{ed}} \in \left[ {1,\infty } \right]$$

2 $${D_e}=\sqrt {\frac{{4({A_d}+{A_0})}}{\pi }}$$

The relative extent of delamination was characterized by a delamination factor independent of the maximum damage diameter. This factor was calculated as the ratio of the equivalent delamination diameter (${D_{_{e}}}$) to the nominal bore diameter (*D*). The equivalent delamination diameter was determined by geometrically approximating the total damaged area, consisting of the delaminated region (${A_d}$) and the drilled hole area (${A_0}$), as a circular region. The maximum and minimum diameters of the delaminated area were denoted as ${D_{_{{\hbox{max} }}}}$ and ${D_{_{{\hbox{min} }}}}$, respectively.

When uncut fibers remain within a drilled hole, they may eventually be compromised by forces and environmental pressures acting on the composite structure over time. As these fibers experience stress, they can be pulled from the matrix, resulting in a degradation of the composite’s integrity and a reduction in its resistance to applied forces. To evaluate the drilling quality of composite materials concerning the presence of uncut fibers, the uncut fiber factor (${F_u}$) is employed. This factor is expressed as a percentage and is calculated by comparing the area of the uncut fibers visible on the surface of the hole to the total area of the hole itself. A lower ${F_u}$ indicates higher drilling quality and better retention of structural integrity, while a higher percentage suggests potential weaknesses that could adversely affect the performance and longevity of the composite material.

3 $${F_u}=\frac{{{A_u}}}{{{A_0}}} \times 100,\,\,\,\,\,\,\,{F_u} \in (0,100]$$

In this equation, ${A_u}$ represents the area of uncut fibers covering the hole’s surface, and ${A_0}$ represents the hole’s area.

## Results and discussion

Table 2 presents the average results of image processing from experiments conducted to investigate the effect of sequential drilling on created delamination and uncut fibers in fabricated composite specimens. These results were obtained through measurements made using ImageJ software, in accordance with the requirements outlined in Eqs. (1)-(3) for both the entrance and exit of the hole.

**Table 2:** Effect of sequential drilling on delamination and uncut fibers in fabricated composite specimens.

Test	Final hole diameter (mm)	Number of drills used	Drill bit diameter (mm)	Feed (mm min^− 1^)	Spindle speed (rpm)	Hole entrance		Hole exit	
						F_ed_	F_u_	F_ed_	F_u_
1	7	1	7	1000	500	1.385	61.197	1.489	38.076
2	7	2	3–7	1000	500	1.374	72.01	1.378	37.523
3	7	3	3–5-7	1000	500	1.313	75.907	1.347	36.785
4	10.1	1	10.1	1000	500	1.321	63.142	1.261	14.107
5	10.1	2	5–10.1.1	1000	500	1.304	65.01	1.257	13.295
6	10.1	3	3–7-10.1	1000	500	1.281	62.704	1.231	1.92

**Fig. 7:**
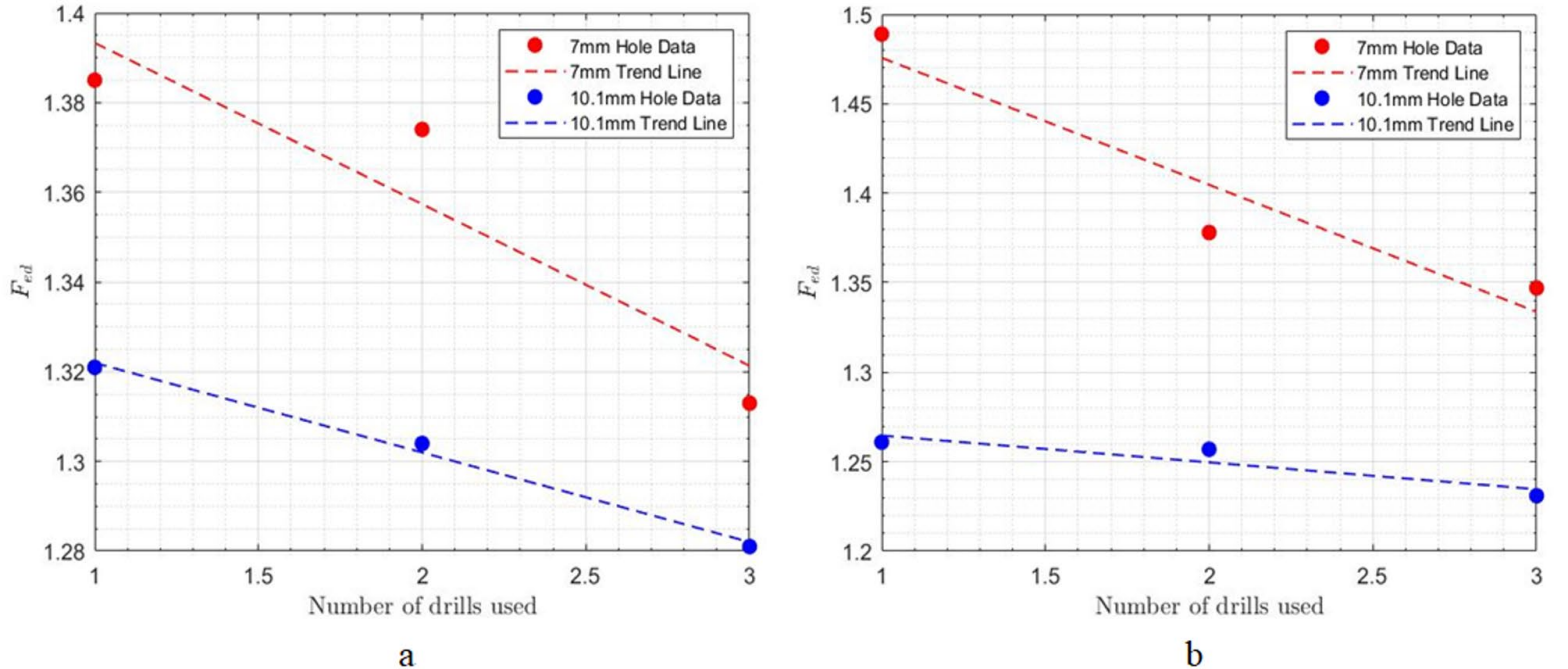
Effect of sequential drilling on the incurred delamination at a feed rate of 1000 mm min^− 1^ and spindle speed of 500 rpm; **a**) hole entrance, **b**) hole exit.

Based on the reported results in Table 2; Fig. 7, it can be observed that sequential drilling in composite materials effectively reduces the amount of delamination at both the entrance and exit of the hole. This reduction can be attributed to the use of smaller drills at the beginning, which create an initial hole and weaken the surrounding composite layers. As a result, less axial force is required to enlarge the hole to the desired diameter with larger drills. Additionally, the presence of this initial hole helps guide the subsequent drills precisely to the location of the hole, thereby significantly reducing vibrations during machining, which further minimizes delamination.

**Fig. 8:**
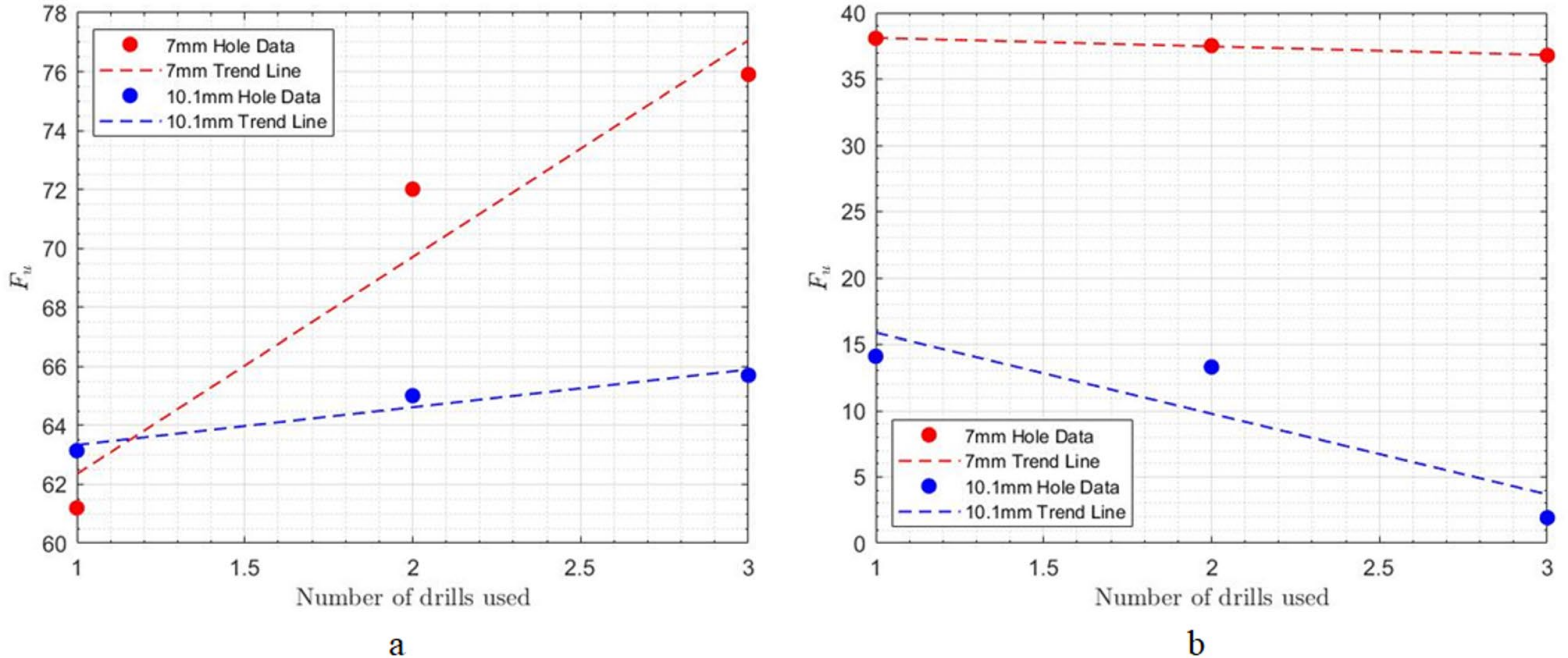
Effect of sequential drilling on the amount of uncut fibers at a feed rate of 1000 mm min^− 1^ and spindle speed of 500 rpm; **a**) hole entrance, **b**) hole exit.

By examining Fig. 8a, it can be inferred that the use of the sequential drilling method, which employs different drills, leads to an increase in the amount of uncut fibers at the entrance of the hole. One reason for this is that pulling the cutting tool out of the workpiece several times, due to the use of multiple drills, causes some fibers at the entrance to be pulled upward. As a result, in the later stages of drilling, many of these fibers become more flexible and are left without proper support from the surrounding material. Consequently, the cutting edge of the drill is unable to make direct contact with these fibers, resulting in a higher number of uncut fibers.

On the other hand, analysis of Fig. 8b reveals that sequential drilling mitigates uncut fiber formation at the hole exit. The progressive weakening of the material around the hole at each drilling stage facilitates the subsequent removal of fibers, even those left uncut in previous stages. The use of incrementally larger drill bits further contributes to the removal of these residual fibers. Reduced axial forces at each step also decrease the probability of fiber fracture and subsequent uncut fiber formation.

**Fig. 9:**
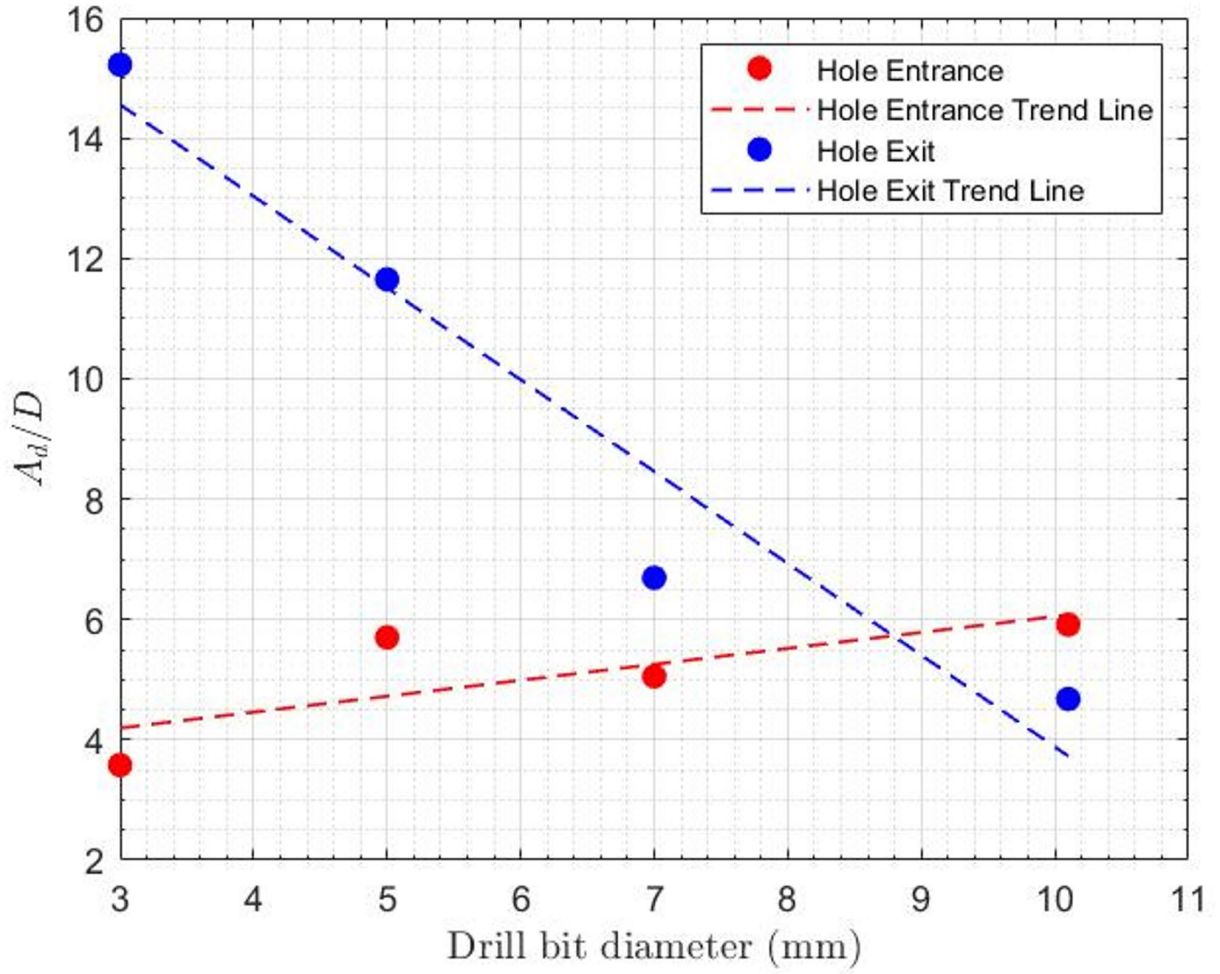
Influence of various drill bit diameter on specimen delamination damage.

Independent of the sequential drilling experiments, a series of holes were drilled using the individual drill bit sizes employed in the sequential process. The resulting delamination area (${A_d}$) at the entrance of each hole was measured and is presented in Table 3; Fig. 9. Analysis of these data revealed a direct positive relationship between drill bit diameter and the ratio of delamination area to hole diameter (${A_d}/D$), confirming that larger drill bit diameters result in greater delamination.

**Table 3:** Influence of various drill bit diameter on specimen delamination damage.

Test	Drill bit diameter (mm)	Hole entrance	Hole exit
		$\frac{{{A_d}}}{D}$	$\frac{{{A_d}}}{D}$
1	3	3.58	15.224
2	5	5.702	11.651
3	7	5.054	6.694
4	10.1	5.915	4.679

This correlation can be attributed to the mechanism of delamination at the entrance of the hole: as the diameter of the drills increases, the spiral flutes on the bits also become larger, providing greater force to separate the composite layers and guide them inward. This increased cutting power leads to a larger delaminated area around the entrance of the hole. Additionally, from Table 3; Fig. 9, it can be observed that as the diameter of the drill bits increases, the amount of delamination (${A_d}$) increases; however, since this increase is not significant relative to the drill bit diameter, the ratio of the delamination area to hole diameter (${A_d}/D$) decreases.

Table 4 presents the results of the experiments conducted to study the effects of varying feed and spindle speeds and to determine their optimal trends during the drilling process, considering the levels of delamination and uncut fibers at the entrances and exits of the holes.

**Table 4:** Effect of varying feed and spindle speed on the incurred delamination and uncut fibers damage during drilling process in fabricated composite specimens.

Test	Drill bit diameter (mm)	Feed (mm min^− 1^)	Spindle speed (rpm)	Hole entrance		Hole exit	
				F_ed_	F_u_	F_ed_	F_u_
1	7	50	1500	1.15	5.666	1.262	24.076
2	7	1000	1500	1.168	20.791	1.267	31.146
3	7	2000	1500	1.26	49.276	1.27	62.151
4	7	1000	500	1.385	61.197	1.489	38.076
5	7	1000	1500	1.258	49.792	1.367	31.146
6	7	1000	3000	1.159	34.8	1.307	29.592
7	10.1	50	1500	1.056	1.281	1.136	4.183
8	10.1	1000	1500	1.167	56.738	1.161	22.486
9	10.1	2000	1500	1.242	70.771	1.196	29.099
10	10.1	1000	500	1.321	63.142	1.261	14.107
11	10.1	1000	1500	1.237	56.738	1.241	12.183
12	10.1	1000	3000	1.166	27.83	1.231	9.349

**Fig. 10:**
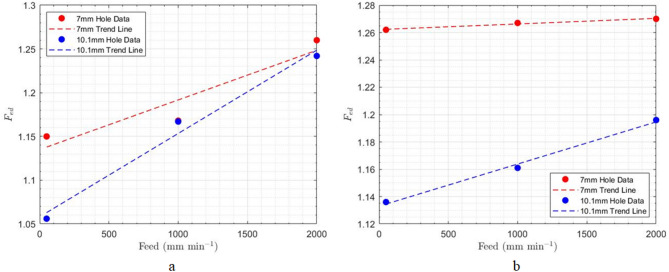
Impact of feed rate on delamination at constant spindle speed of 1500 rpm; **a**) hole entrance, **b**) hole exit.

According to the results of the experiments and as shown in Table 4; Fig. 10, it can be concluded that increasing feed rates lead to a higher amount of delamination at both the entrance and exit of the hole. This finding can be justified by noting that as feed increases, the axial force applied to the workpiece also increases, which aligns with the direct relationship between axial force and delamination. Additionally, higher feed rates result in increased vibrations when the cutting tool enters the workpiece, and at elevated feed rates, the cutting temperature also rises significantly.

**Fig. 11:**
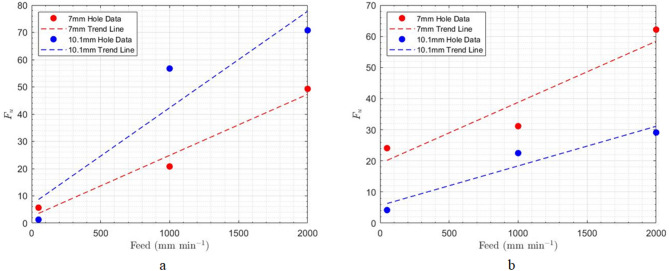
Impact of feed rate on uncut fibers at constant spindle speed of 1500 rpm; (a) hole entrance, (**b**) hole exit.

Regarding uncut fibers, the amount increases at both the entrance and exit of the holes as feed rates increase (see Fig. 11). One reason for this trend is that, with higher feed rates, the cutting tool does not have sufficient time to cut the composite reinforcing fibers, resulting in a greater number of uncut fibers. Additionally, increasing the feed rate raises the axial force applied to the composite, which alters the orientation of the fibers located in the drilling direction. As a result, these fibers can be displaced away from the drilling path and remain uncut.

**Fig. 12:**
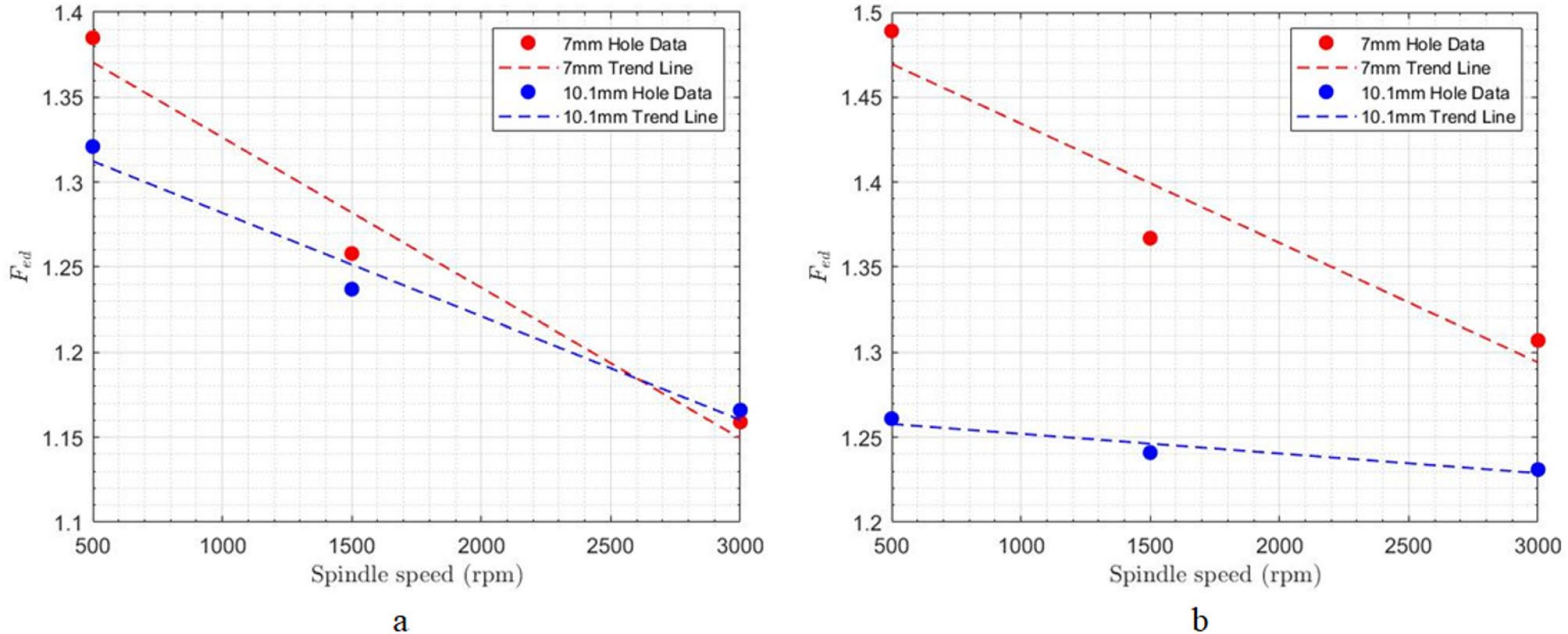
Impact of spindle speed on delamination at constant feed rate of 1000 mm min^− 1^; **a**) hole entrance, **b**) hole exit.

At a constant feed rate, a significant inverse correlation was observed between spindle speed and the delamination factor at both the entrance and exit of the drilled holes (Table 4; Fig. 12). This reduction in delamination is attributed to the increased rotational speed of the cutting tool. Higher spindle speeds lead to a greater number of cutting engagements within a given material volume. Consequently, fibers that remain uncut at lower spindle speeds, delaminating due to axial forces, are more effectively severed at higher speeds, thus reducing the extent of delamination.

**Fig. 13:**
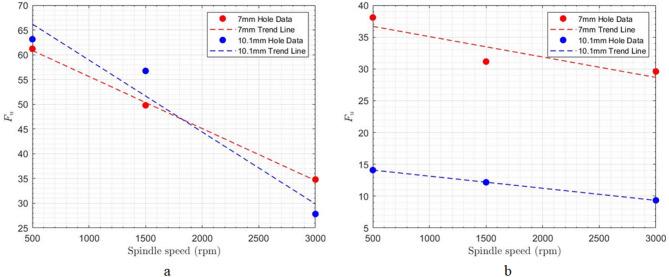
Impact of spindle speed on uncut fibers at constant feed rate of 1000 mm min^− 1^; **a**) hole entrance, **b**) hole exit.

Also, as shown in Fig. 13, increasing the cutting speed reduces the number of uncut fibers at the hole entrance and exit. This is attributed to the increased frequency of fiber engagement with the cutting tool’s edge at higher speeds, resulting in more complete fiber severance.

**Fig. 14:**
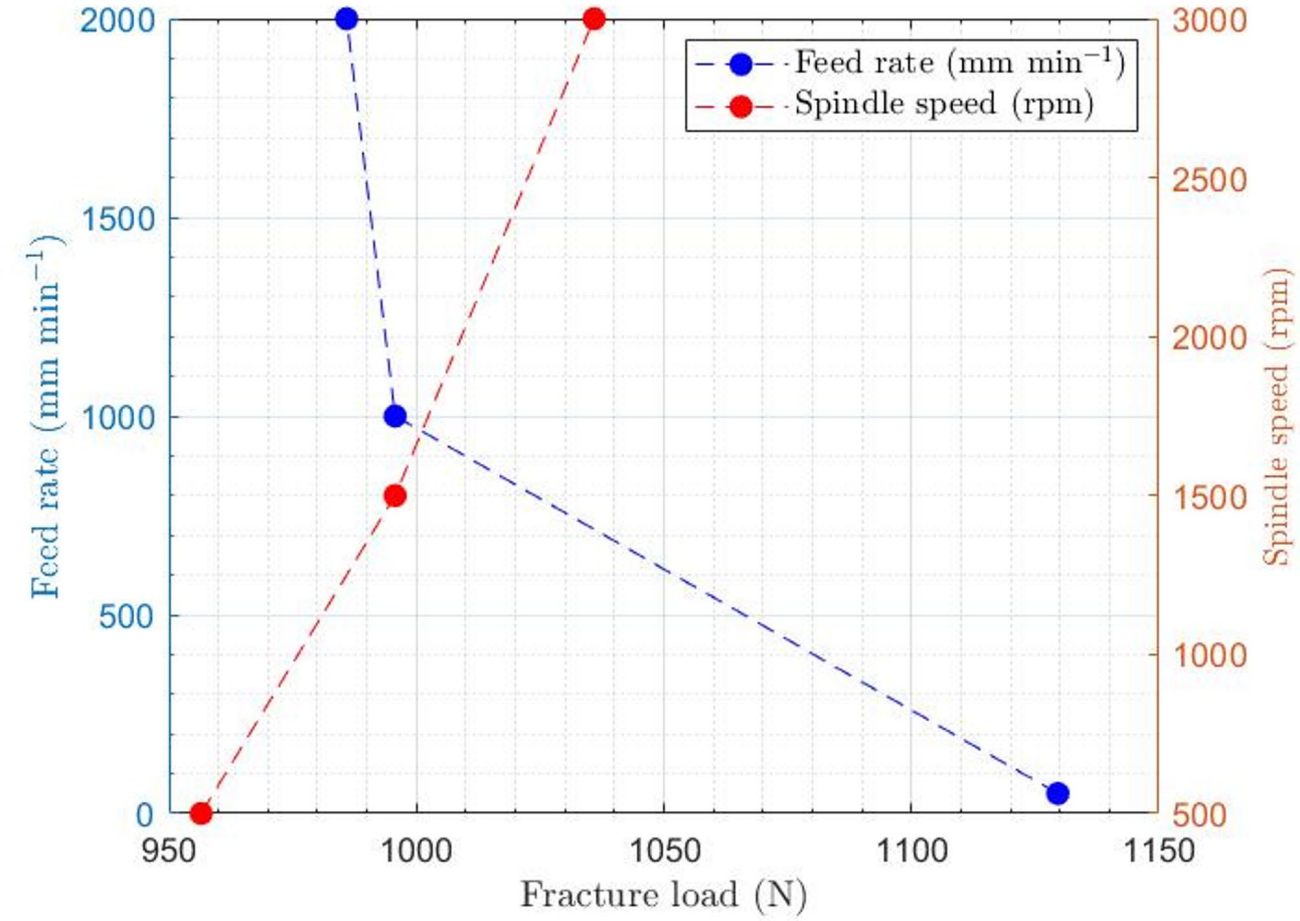
Effect of various feed rate and spindle speed on the three-point bending force required to break drilled specimens.

According to Table 5; Fig. 14, which present the results of the three-point bending test on drilled specimens with various feed rates and spindle speeds, it is evident that increasing the feed rate leads to greater delamination damage around the holes, resulting in a reduced force required to achieve failure in these specimens. Conversely, as the rotational speed of the cutting tool increases, the forces required to break the specimens during the bending test also increase, due to the reduced amount of delamination damage associated with higher rotational speeds.

**Table 5:** Effect of various feed rate and spindle speed on the three-point bending force required to break drilled specimens.

Test	Hole diameter (mm)	Feed rate (mm min^− 1^)	Spindle speed (rpm)	Fracture load (*N*)	Hole entrance		Hole exit	
					F_ed_	F_u_	F_ed_	F_u_
1	7	50	1500	1129.6	1.15	5.666	1.262	24.076
2	7	1000	1500	995.7	1.168	20.791	1.267	31.146
3	7	2000	1500	985.9	1.26	49.276	1.27	62.151
4	7	1000	500	956.5	1.385	61.197	1.489	38.076
5	7	1000	1500	995.7	1.258	49.792	1.367	31.146
6	7	1000	3000	1035.9	1.159	34.8	1.307	29.592

**Fig. 15:**
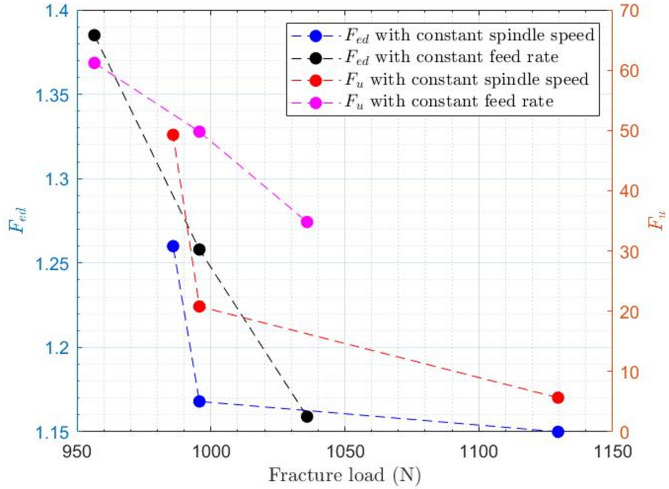
Correlation between entrance-side damage parameters and laminate fracture load under different drilling conditions.

As shown in Fig. 15, both the delamination factor (${F_{ed}}$) and uncut fiber factor (${F_u}$) measured at the hole entrance exhibit a decreasing trend with increasing fracture load, indicating that specimens with lower entrance damage can sustain higher bending loads. The reduction in ${F_{ed}}$ reflects improved interlaminar integrity, while the decrease in ${F_u}$ suggests more effective fiber cutting and better load transfer capability. Under constant spindle speed, the decline in damage factors is gradual, whereas at constant feed rate, the reduction is more pronounced, emphasizing the stronger influence of feed rate on entrance-side damage and, consequently, on the mechanical performance of the composite laminate.

**Fig. 16:**
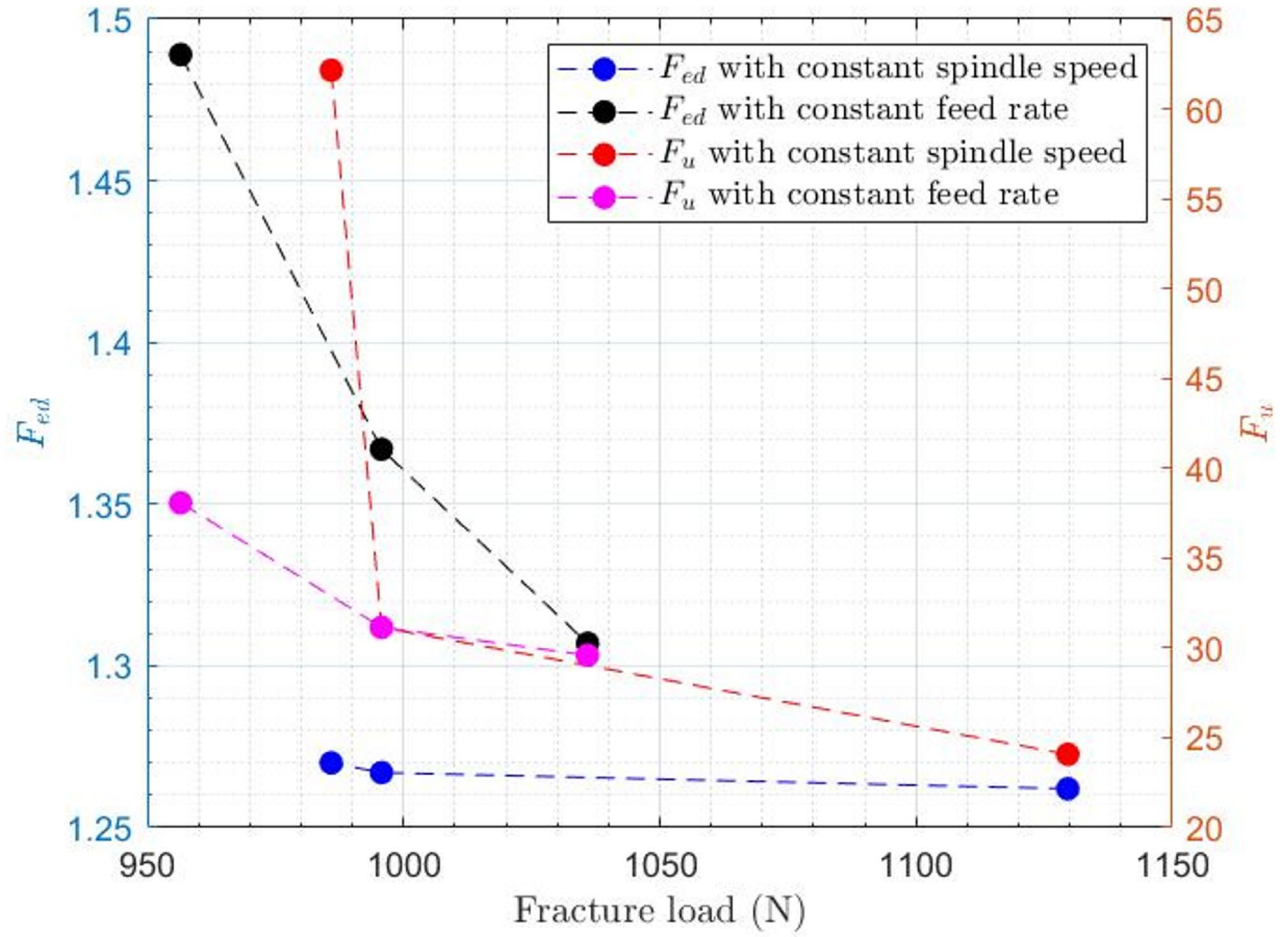
Correlation between exit-side damage parameters and laminate fracture load under different drilling conditions.

Figure 16 illustrates the correlation between the fracture load from three-point bending tests and two key exit-hole damage parameters. The data reveals a definitive inverse relationship, where an increase in fracture load is consistently associated with a decrease in both damage factors. This trend demonstrates that the structural performance of the laminate under bending load is highly sensitive to the quality of the hole exit. Lower exit-side damage, characterized by less delamination (lower ${F_{ed}}$) and cleaner fiber cutting (lower ${F_u}$), directly translates to a higher load-bearing capacity.

Furthermore, the machining strategy significantly influences this relationship. For both damage factors, the “constant feed rate” condition results in a steeper decline in damage with increasing load compared to the “constant spindle speed” condition. This suggests that maintaining a constant feed rate is a more effective strategy for mitigating exit-side damage and preserving the mechanical integrity of the composite, as the benefits are more substantial for a given increase in fracture load.

## Conclusion

In this study, the effects of sequential drilling and effective machining parameters on the quality of drilled holes in composite materials were investigated, focusing on damages such as delamination and uncut fibers. The key findings of this study are as follows:

- The use of multiple drills consecutively, progressing from smaller sizes to the desired size, leads to a reduction in delamination at both the entrance and exit of the hole. This reduction is attributed to the gradual distribution of cutting load and lower peak thrust forces (axial force), which reduce tool-induced vibrations and stress concentration around the hole boundary.
- The sequential drilling method results in an increase in the amount of uncut fibers at the entrance of the hole, caused by the repeated pulling of the cutting tool out of the workpiece. However, experimental results show that this method reduces the amount of uncut fibers at the exit of the hole.
- The observed trends suggest that sequential drilling decreases instantaneous cutting resistance, effectively mitigating drilling-induced damage even without direct thrust or torque measurement.
- The results highlight that careful control of feed rate and spindle speed, combined with the sequential drilling strategy, can optimize the trade-off between drilling efficiency and hole integrity in composite manufacturing.
- The experiments indicate that increasing the diameter of the drill bit directly affects the amount of delamination around the entrance of the hole, while it has the opposite effect on the delamination around the hole exit.
- Furthermore, increasing the diameter of the drill correlates with an increase in the amount of uncut fibers at both the entrance and exit of the hole.
- Increasing the feed rate leads to greater delamination at both the entrance and exit of the hole due to the increased axial force applied by the cutting tool to the workpiece.
- Conversely, increasing the spindle speed reduces delamination at both the entrance and exit of the hole, as the cutting edge of the tool rotates more frequently within a given time frame.
- Reducing the feed rate increases the force required to break the workpiece during the bending process because of the decreased amount of delamination.
- In contrast, reducing the spindle speed lowers the force required to break the workpiece during the bending process due to an increased level of delamination.
- Increased feed rates correspond to a higher amount of uncut fibers at both the entrance and exit of the hole, attributed to insufficient time for the cutting tool to cut the reinforcing fibers effectively.
- Conversely, as spindle speed increases, the amount of uncut fibers at both the entrance and exit of the hole decreases.
- The analysis of both entrance and exit-hole damage demonstrates that the feed rate exerts a more significant influence on the induced drilling damage than the spindle speed.

## Supplementary Information

Below is the link to the electronic supplementary material.

Supplementary Material 1

## Data Availability

The data supporting the findings of this study are included in the manuscript and its supplementary information.

## Abbreviations

CFRPs: Carbon fiber-reinforced polymers
MQL: Minimum quantity lubrication
RUM: Rotary ultrasonic machining
3PB: Three-point bending
HSS: High-speed steel
FGMs: Functionally graded materials
${F_{ed}}$: Equivalent delamination factor
$${Q_d}$$: Quality function
${A_d}$: Delaminated area around the hole
${D_{_{\max }}}$: Maximum damage diameter
${D_e}$: Equivalent delamination diameter
*D*: Bore diameter
${A_0}$: Bore hole area
${F_u}$: Uncut fiber factor
${A_u}$: The area covered by uncut fibers
$${F_a}$$,$${F_{da}}$$: Delamination factor
