# Supplementary material for: The influence of sequential drilling and machining parameters on uncut fiber formation and delamination damage in fiber-reinforced composites
Source: Sci Rep. 2026 Feb 21;16:10132. doi: 10.1038/s41598-026-40786-y (PMC13022421; doi:10.1038/s41598-026-40786-y)
Supplement: Supplementary file 1 — Supplementary Material 1 [file 41598_2026_40786_MOESM1_ESM.docx]

Highlights

- A comprehensive investigation into the effects of sequential drilling methodologies on damage formation in fiberglass composites, specifically focusing on varying drill diameters.
- Analysis of critical machining parameters—feed rate and spindle speed—and their influence on incurred damage during the drilling process.
- Implementation of image processing techniques to quantify damage by analyzing hole geometry, with particular emphasis on uncut fibers and delamination at drill hole entrances and exits.
- Utilization of three-point bending tests (3PB) to evaluate the fracture load of drilled specimens, correlating the extent of observed damage with mechanical performance.
